# Integrative multi-omics analysis reveals a gut-liver axis signature of metabolic reprogramming associated with response to transarterial chemoembolization in hepatocellular carcinoma

**DOI:** 10.1186/s12876-026-04978-0

**Published:** 2026-06-17

**Authors:** Xiaojing Ren, Zhenyu Liu, Yifan Wang, Peng Guo, Shuyao Cheng, Ruiping Zhang

**Affiliations:** 1 Third Hospital of Shanxi Medical University, Shanxi Bethune Hospital, Shanxi Academy of Medical Sciences, Tongji Shanxi Hospital, Taiyuan, 030032 China; 2 Clinical College of Xiangnan University, Chenzhou, 423000 China; 3 School of Civil and Commercial Law, Southwest University of Political Science and Law, Chongqing, 401120 China; 4 School of Basic Medical Sciences, Shanxi Medical University, Shanxi, 030600 China; 5 The Radiology Department of Shanxi Provincial People’s Hospital Affiliated to Shanxi Medical University, Taiyuan, 030001 China

**Keywords:** Hepatocellular Carcinoma, Transarterial Chemoembolization, Metabolomics, Transcriptomics, Gut-Liver Axis, Bile Acid Metabolism, Tryptophan Metabolism, Biomarker

## Abstract

**Background:**

Transarterial chemoembolization (TACE) is a standard treatment for intermediate-stage hepatocellular carcinoma (HCC), but response is highly variable. The systemic metabolic impact of TACE and its connection to tumor-intrinsic factors governing efficacy remain poorly understood. We hypothesized that by comparing host fecal metabolomics with tumor transcriptomics, we could identify convergent pathways suggestive of a gut-liver axis signature of TACE response.

**Methods:**

We performed untargeted fecal metabolomics in a prospective paired cohort of 30 HCC patients sampled before TACE and again on post-TACE day 4, an early time point selected to capture acute ischemic, inflammatory, and metabolic perturbations after embolization. To characterize tumor-intrinsic programs associated with efficacy, we analyzed the independent public transcriptomic dataset GSE104580 containing pre-treatment tumors from TACE responders and non-responders. Cross-cohort integration was conducted at the pathway level to identify convergent biological themes. For metabolomics, paired univariate testing with Benjamini–Hochberg false discovery rate (FDR) correction and Variable Importance in Projection (VIP) > 1 from PLS-DA were used; for transcriptomics, differential expression used adjusted p < 0.05 and |log2FC|> 1, followed by GSEA.

**Results:**

TACE induced a shift in the fecal metabolome, with significant enrichment of glycerophospholipid metabolism, together with changes in bile acid-related metabolites and tryptophan/vitamin B6-related metabolites. Lysophosphatidylcholines (LysoPCs), including LysoPC (22:4), and bile acid-related metabolites were increased after TACE, consistent with acute treatment-associated tissue metabolic perturbation. Independent transcriptomic analysis revealed that responder tumors were enriched for primary bile acid biosynthesis, fatty acid degradation, and tryptophan metabolism, with higher expression of CYP7A1, ACOX1, IDO1, and TDO2, whereas non-responders were enriched for Cell cycle, DNA replication, and ribosome-related programs. Because the metabolomics and transcriptomics datasets were derived from separate cohorts, these convergent findings support a pathway-level cross-cohort model rather than direct patient-level tumor-fecal linkage.

**Conclusion:**

Our comparative analysis suggests a potential gut-liver axis signature associated with TACE efficacy. We therefore present fecal LysoPC as a candidate non-invasive pharmacodynamic readout of acute post-TACE tissue injury and host-microenvironmental perturbation rather than a tumor-necrosis-specific biomarker. Likewise, the convergence on bile acid and tryptophan metabolism suggests that a responder-associated tumor metabolic phenotype may be relevant to TACE sensitivity. These findings offer potential non-invasive biomarkers and highlight new therapeutic targets to enhance TACE efficacy.

**Supplementary Information:**

The online version contains supplementary material available at 10.1186/s12876-026-04978-0.

## Introduction

Hepatocellular carcinoma (HCC) is the sixth most common malignancy and the third leading cause of cancer-related mortality worldwide [1]. For patients with unresectable, intermediate-stage disease (BCLC stage B) [2], transarterial chemoembolization (TACE) is a cornerstone of therapy, aiming to induce tumor necrosis by delivering a combination of locoregional chemotherapy and embolic agents [2–5]. Despite its widespread use, the clinical outcomes of TACE are highly heterogeneous [6, 7]. A significant proportion of patients fail to achieve an objective response, and many experience rapid disease progression, underscoring an urgent clinical need for robust biomarkers to predict therapeutic efficacy and guide personalized treatment strategies [8–10]. Current prognostic tools, which rely heavily on baseline tumor characteristics like size and number, vascular invasion, and serum alpha-fetoprotein (AFP) levels, often fall short in capturing the complex biological determinants of treatment response [11, 12]. This highlights the necessity of exploring deeper molecular and systemic mechanisms that govern TACE sensitivity.

The liver does not function in isolation; it is intricately connected with the gastrointestinal tract through a bidirectional communication network known as the gut-liver axis. This axis involves the constant interplay between the gut microbiota, the integrity of the intestinal barrier, and hepatic function [13, 14]. Metabolites produced by the gut microbiota, such as secondary bile acids [15], short-chain fatty acids (SCFAs) [13, 16], and components like lipopolysaccharide (LPS) [17, 18], are transported via the portal vein directly to the liver. These molecules can profoundly influence hepatic processes, including inflammation, lipid metabolism, fibrosis, and ultimately, carcinogenesis [19, 20]. A growing body of evidence indicates that gut dysbiosis-an imbalance in the microbial community-is closely associated with the progression of chronic liver diseases and the development of HCC [21]. Therefore, the metabolic output of the gut, which can be non-invasively sampled from feces, represents a valuable window into the systemic physiological state and its influence on liver pathology and therapeutic response.

Understanding the variability in TACE response requires a systems-level perspective that integrates the local tumor biology with the systemic host response. A multi-omics approach is well suited for this challenge. Fecal metabolomics provides a non-invasive, functional readout of the host's metabolic state and the activity of the gut-liver axis, reflecting the combined metabolic output of the host and its microbiome. Concurrently, tumor transcriptomics offers a high-resolution map of the biological pathways active within the cancer cells and the tumor microenvironment. In the present study, these two data layers were not obtained from the same individuals; instead, we compared paired fecal metabolomic changes observed in a local TACE cohort with pre-treatment tumor transcriptomic programs associated with TACE response in an independent public cohort. Accordingly, the integrative component of this work was designed to identify convergent pathway-level biological themes and generate testable hypotheses regarding biomarkers and mechanisms of TACE response in HCC, rather than to establish direct patient-level tumor–fecal crosstalk.

## Materials and methods

### Study design and patient cohorts

#### Metabolomics cohort

A prospective, single-center, self-controlled study was designed to investigate the effects of TACE on the fecal metabolome. The cohort comprised 30 patients diagnosed with primary HCC who were scheduled to receive their first TACE treatment at Shanxi Bethune Hospital between November 2023 and December 2024. The study protocol was approved by the Institutional Ethics Committee of Shanxi Bethune Hospital, and all participants provided written informed consent in accordance with the Declaration of Helsinki. This paired within-patient design was intended to reduce inter-individual variability. Baseline clinicopathological features indicate a relatively homogeneous cohort (mean age 58.7 ± 9.2 years; 25/30 male; 24/30 HBV-related HCC; 28/30 Child–Pugh A; 22/30 BCLC stage B), which partially reduces disease-related heterogeneity.

Inclusion criteria were: (1) diagnosis of primary HCC confirmed by pathology or clinical imaging (meeting AASLD or EASL criteria); (2) scheduled for first-ever TACE; (3) no use of antibiotics, probiotics, or prebiotics within one month prior to treatment; and (4) ability to provide informed consent and comply with sample collection protocols. Exclusion criteria included: (1) presence of other malignancies; (2) known inflammatory bowel disease or other severe gastrointestinal disorders; (3) severe cardiac, pulmonary, or renal dysfunction; (4) pregnancy or lactation; and (5) inability to provide adequate fecal samples. Patient-level clinical records additionally contained variables such as BMI, alcohol history, smoking history, family history, disease history, and routine laboratory indices, which were manually reviewed to assess cohort heterogeneity; however, these variables were not prospectively structured as a formal multivariable covariate matrix for metabolomics modeling. Therefore, the observed fecal metabolic shifts should be interpreted as TACE-associated perturbations observed under a partially controlled clinical design rather than as proof that every metabolite change was exclusively attributable to TACE itself.

## Transcriptomics cohort

Tumor transcriptomic data were downloaded from the Gene Expression Omnibus (GEO) database (accession number: GSE104580). This dataset contains profiles from pre-treatment tumor tissues of HCC patients classified as TACE responders or non-responders.

## Sample collection and processing

### Fecal samples

For the metabolomics cohort, fresh fecal samples were collected from each of the 30 patients at two time points: one day before the TACE procedure (Pre-operation) and on the fourth day after the procedure (Post-operation). Patients were instructed to collect 2–5 g of mid-stream stool using sterile collection tubes. Samples were transported to the laboratory within 2 h of collection and immediately stored at −80 °C to prevent metabolite degradation until analysis. Post-TACE day 4 was selected to capture the early acute metabolic consequences of embolization, tumor ischemia, tissue injury, and inflammatory activation; however, this time point represents an early pharmacodynamic snapshot rather than a stable long-term metabolic endpoint.

## Fecal metabolomics analysis

Frozen fecal samples (50 mg) were homogenized in a pre-chilled 80% methanol solution containing a mixture of internal standards for data normalization. Homogenization was performed using a high-throughput tissue grinder at low temperatures. The resulting homogenate was centrifuged at high speed, and the supernatant, containing the extracted metabolites, was collected for analysis.

## MALDI-TOF MS analysis

MALDI-TOF MS was chosen for this initial exploratory analysis due to its high-throughput capability and sensitivity for certain classes of small molecules, including lipids, which were anticipated to be significantly altered by TACE. Metabolite extracts were mixed with a carbon nanomaterial-based MALDI matrix solution and spotted onto a MALDI target plate. After drying, the plate was inserted into a MALDI-TOF mass spectrometer. Data were acquired in both positive and negative ion modes to ensure broad coverage of the metabolome. The instrument parameters, including laser energy, voltage, and detector settings, were optimized for high-resolution mass spectral acquisition.

## Data pre-processing

The raw mass spectrometry data underwent a rigorous pre-processing pipeline. This included peak identification and extraction, baseline correction to remove noise, peak alignment across all samples to correct for minor mass shifts, and data normalization using the internal standards to account for variations in sample preparation and instrument response. Detailed lists of key differential metabolites are provided in Table 1.

**Table 1:** Differential fecal metabolites in paired pre-TACE and post-TACE day 4 samples identified by untargeted MALDI-TOF MS. Regulation direction, log2 (fold change), and VIP score are shown for metabolites detected in negative- and positive-ion modes

**Neg-Metabolite**	**Regulation**	**Log2 (Fold Change)**	**VIP Score **
7-Methylxanthine	Up	6.89019209	3.32509629
N-Acetyl-L-phenylalanine	Up	6.28490860	2.17563341
Taurodeoxycholic acid	Up	3.00002585	1.71450319
LysoPE(18:0/18:1(9Z/12Z))	Up	2.37464775	1.86314751
LysoPE(22:4(7Z,10Z,13Z,16Z)/0:0)	Up	2.23981486	2.32319395
15-hydroxyculmorin	Up	1.91713321	1.88465586
Pyridoxal 5'-phosphate	Up	1.87075521	1.67308424
Tetrahydroindol 1	Down	-1.24910243	1.65995442
5-Acetyl-2,3-dihydro-1,4-thiazine	Down	-1.28846636	2.11984981
PG(22:6(4Z,7Z,10Z,13Z,16Z,19Z))	Down	-1.29003500	1.78441192
Methyl 3-indolyacetate	Down	-1.31291936	1.69956355
Lactapiperol C	Down	-1.32822723	2.20312196
Dehydroabietic acid	Down	-1.34067538	1.03852904
Octadeca-2,4-dienedioic acid	Down	-1.37256546	1.76127947
beta-Estradiol 17-Acetate	Down	-1.37511741	2.38923888
3-(1-cyano-1,2-dihydroisoquinolin-2-yl)-3-oxopropyl propionate	Down	-1.37969712	1.68573189
(-)-Methylenelactocin	Down	-1.38084628	2.39080109
2-Phenylethanol glucuronide	Down	-1.44946080	1.49258033
Cyclo(4-hydroxy-R-Pro-S-Leu)	Down	-1.45960311	1.91889601
Glycoursodeoxycholic acid	Down	-1.48236748	2.52717266
Enterolactone 3'-glucuronide	Down	-1.59420417	1.17587742
LysoPE(20:5(5Z,8Z,11Z,14Z,17Z)/0:0)	Down	-1.66878725	1.35697082
7-Ketolithocholic acid	Down	-1.69908569	1.33954238
N-Methylcimasinadane	Down	-1.88122829	1.93916324
8-Mercaptooctanoic acid	Down	-1.89804402	1.49063369

**Pos-Metabolite**	**Regulation**	**Log2 (Fold Change)**	**VIP Score**
Nervonic ceramide	Up	9.46868313	3.03015772
Finerenone	Up	9.15837480	1.53349547
(-)-Chrysogine	Up	6.72678513	1.82445598
N-Carbamoyl-2-amino-2-(4-hydroxyphenyl)acetic acid	Up	2.65439190	1.87690916
LysoPC(22:4(7Z,10Z,13Z,16Z)/0:0)	Up	2.35767887	1.83081503
11(Z),14(Z),17(Z)-Eicosatrienoic acid	Up	2.20106293	0.82720925
PC(20:3(5Z,8Z,11Z)/20:3(8Z,11Z,14Z))	Up	2.13673252	0.74201428
Adenosine-2-monophosphate	Up	2.07727574	2.91795730
Arachidic Acid	Up	1.82054433	91795731
N6-Me-Adenosine	Up	1.79970533	2.16410162
( ±)-trans-Acenaphthene-1,2-diol	Up	1.79562938	2.06776409
Creatine	Up	1.70502066	0.27606572
PC(P-16:0/22:4(7Z,10Z,13Z,16Z))	Up	1.64316362	0.63164511
Isopropalin	Up	1.62894379	2.30357632
3-Hydroxy-3-methylbutanoic acid	Up	1.57781896	1.48450724
Lauroyl diethanolamide	Up	1.52659030	0.81485798
Val Pro Phe	Up	1.50078166	1.26589245
Nitrofurazone	Up	1.47434054	1.01970843
1-beta-D-glucopyranosyl-L-tryptophan	Up	1.44186532	1.64379581
SM(d18:0/18:1(9Z))	Up	1.43252885	0.81663511
Lanrylamide E	Down	-1.15957532	1.84412917
Elaeokanine C	Down	-1.15974475	1.36991680
Sodium [dodecanoyl(methyl)amino]acetate	Down	-1.16033237	1.88412216
Nigrolineabiphenyl A	Down	-1.17199176	0.83191368
Isobutylpyramine (C4)	Down	-1.19741256	1.08549774
Nepodin	Down	-1.20517091	2.17119186
Lithocholic	Down	-1.22079656	1.38471483
Methyl indole-3-acetate	Down	-1.27017475	1.98926402
Tiglyl carnitine	Down	-1.27086154	1.90282790
2,4-dihydroxyheptadec-16-en-1-yl acetate	Down	-1.27697926	1.21952260
Enterolactone	Down	-1.29300758	2.21940135
Sananene	Down	-1.34488212	2.57154477
5-Hydroxyindole-3-acetic acid	Down	-1.34625457	2.81811988
Triptolide	Down	-1.41997882	1.25720955
1,2-dihydroheptadec-16-yn-1-yl acetate	Down	-1.48950503	1.35993573
Practafol	Down	-1.90464475	1.92138364
Melophin M	Down	-1.91692860	1.42181947
Eupatorin	Down	-1.98629969	1.63747768
3,4,5-Trimethoxyphenyl(acetic acid)	Down	-2.18924648	.85836943

## RNA sequencing and bioinformatic analysis

Gene expression levels were quantified as transcripts per million (TPM) or raw counts. Differential expression analysis between the Response and Non-Response groups was performed using the limma package (v3.56.2) in R, with a threshold of an adjusted *p*-value < 0.05 and |log2(Fold Change)|> 1. Gene Set Enrichment Analysis (GSEA) was subsequently performed using the clusterProfiler package (v4.8.3) with the KEGG pathway gene sets to identify significantly enriched biological pathways.

## Validation of metabolic signatures in public cohorts

To validate the clinical relevance of the identified metabolic signatures, publicly available transcriptomic and clinical data were obtained for two independent HCC cohorts: The Cancer Genome Atlas Liver Hepatocellular Carcinoma (TCGA-LIHC, *n* = 368) and GSE14520 (*n* = 242). For each tumor sample, enrichment scores for the primary bile acid biosynthesis and tryptophan metabolism pathways were calculated using the single-sample gene set enrichment analysis (ssGSEA) algorithm. The association between pathway enrichment scores and clinical-pathological features (histological grade, BCLC stage, pathological stage) was assessed using the Kruskal–Wallis test. To evaluate prognostic significance, patients in each cohort were stratified into “High” and “Low” enrichment groups based on the median pathway score. Overall survival (OS) differences between these groups were visualized using Kaplan–Meier curves, and statistical significance was determined using the log-rank test. A *p*-value < 0.05 was considered statistically significant.

## Statistical analysis

All statistical analyses were performed using R software (v4.2.1). For the metabolomics data, unsupervised multivariate analysis was conducted using Principal Component Analysis (PCA) to visualize overall data structure and identify outliers. Supervised multivariate analysis was performed using Partial Least Squares Discriminant Analysis (PLS-DA) to maximize the separation between pre- and post-TACE groups and identify key differentiating metabolites. Metabolites with a VIP score greater than 1 were considered significant contributors to the model. Univariate statistical tests (e.g., paired t-test or Wilcoxon signed-rank test) with Benjamini–Hochberg False Discovery Rate (FDR) correction were used to identify significantly altered individual metabolites. Statistical tests are specified more explicitly in the results and figure legends. The representative post-TACE fecal metabolic alterations are detailed in Table 1, and the baseline clinicopathological profile of the 30-patient metabolomics cohort is summarized in Table 2.

**Table 2:** Baseline clinicopathological characteristics of the metabolomics cohort. This table provides a comprehensive overview of the patient population from which the metabolomics data were derived, establishing the clinical context of the findings. The cohort primarily consists of male patients with HBV-related, intermediate-stage HCC and well-preserved liver function

Characteristic	Value (n = 30)
Age, years (mean ± SD)	58.7 ± 9.2
Sex (Male, n (%))	25 (83.3%)
Etiology, n (%)	
HBV	24 (80.0%)
HCV	2 (6.7%)
Alcohol	3 (10.0%)
Other/Unknown	1 (3.3%)
Child–Pugh Class, n (%)	
A	28 (93.3%)
B	2 (6.7%)
BCLC Stage, n (%)	
A	5 (16.7%)
B	22 (73.3%)
C	3 (10.0%)
Tumor Number (median)	2 [1-4]
Largest Tumor Diameter, cm (mean ± SD)	6.5 ± 2.8
Baseline AFP, ng/mL (median)	150 [35–800]

## Results

### TACE profoundly remodels the fecal metabolic landscape in HCC Patients

**Fig. 1:**
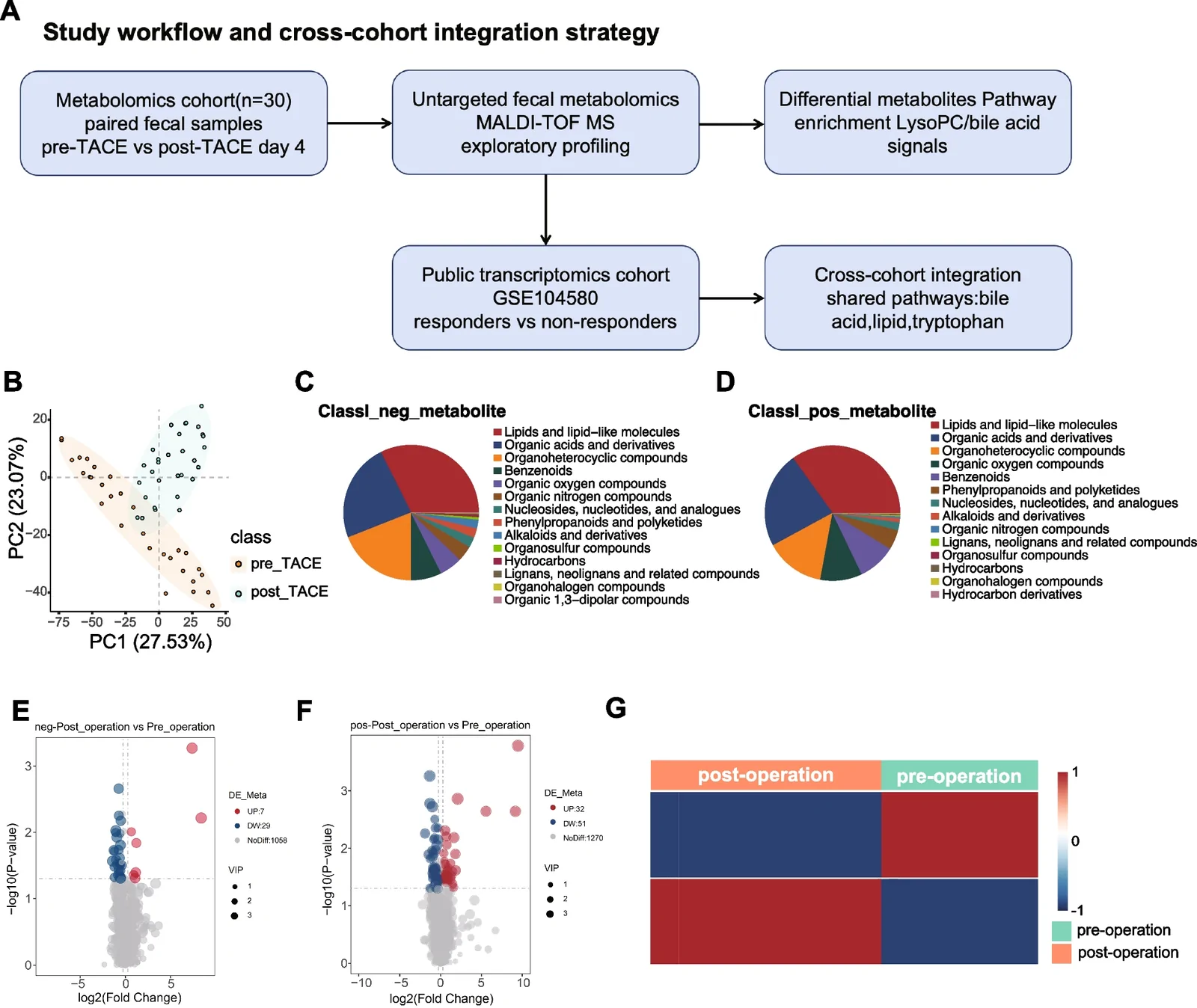
TACE profoundly remodels the fecal metabolic landscape in HCC patients. **A** Schematic overview of the study workflow, including the metabolomics cohort, transcriptomics cohort, and cross-cohort integration strategy. **B** PCA plot of fecal metabolic profiles from 30 HCC patients, showing distinct clustering of pre-operation (orange) and post-operation (teal) samples. **C**, **D** Chemical classification of all identified metabolites in negative (**C**) and positive (**D**) ion modes, highlighting the predominance of lipids and lipid-like molecules. **E**, **F** Volcano plots illustrating differentially abundant metabolites between post- and pre-operation samples in negative (**E**) and positive (**F**) ion modes. Red and blue dots represent significantly upregulated and downregulated metabolites, respectively. **G** Heatmap of the differentially abundant metabolites demonstrating clear separation and distinct metabolic signatures between pre- and post-operation groups

To facilitate interpretation of the study design, we first summarized the overall workflow and cross-cohort integration strategy in Fig. 1A. Briefly, paired fecal samples collected from 30 patients with HCC before TACE and on post-TACE day 4 were subjected to untargeted exploratory metabolomic profiling using MALDI-TOF MS to identify treatment-associated metabolic alterations, including differential metabolites and enriched pathways. In parallel, a public pre-treatment tumor transcriptomic cohort (GSE104580) was analyzed to compare TACE responders and non-responders and to identify response-associated transcriptional programs. The two independent data layers were then integrated at the pathway level to determine whether convergent biological processes were shared across cohorts. This workflow identified cross-cohort concordance in bile acid metabolism, lipid remodeling, and tryptophan metabolism, thereby providing a hypothesis-generating framework for the proposed gut-liver axis signature associated with TACE response.

To assess the systemic metabolic impact of TACE, we first performed a global, non-targeted analysis of fecal metabolites from 30 HCC patients before and after treatment. The baseline clinicopathological characteristics of these patients are summarized in Table 2. PCA of the complete metabolic profiles revealed a distinct and significant separation between the pre-operation and post-operation samples (Fig. 1B) indicating that TACE induces a rapid and profound shift in the host's systemic metabolic state as reflected in the gut environment. To understand the composition of the fecal metabolome in this cohort, we performed chemical classification of all detected features. In both negative and positive ion modes, the most abundant chemical classes were lipids and lipid-like molecules, organic acids and their derivatives, and organoheterocyclic compounds (Fig. 1C, D). This establishes that lipid and organic acid metabolism are dominant components of the fecal metabolic landscape in HCC patients. Differential analysis identified a large number of metabolites that were significantly altered following TACE. Volcano plots for both negative and positive ion modes demonstrate that multiple metabolic features were either significantly upregulated or downregulated in the post-operation samples compared to baseline (Fig. 1E, F). This metabolic reprogramming was further visualized by a heatmap of the most significantly altered metabolites. The heatmap showed distinct clustering of the pre-operation and post-operation samples into two separate groups, reinforcing the PCA findings and confirming the robustness of the TACE-induced metabolic signature (Fig. 1G).

## Glycerophospholipid and bile acid metabolism are key pathways altered post-TACE

**Fig. 2:**
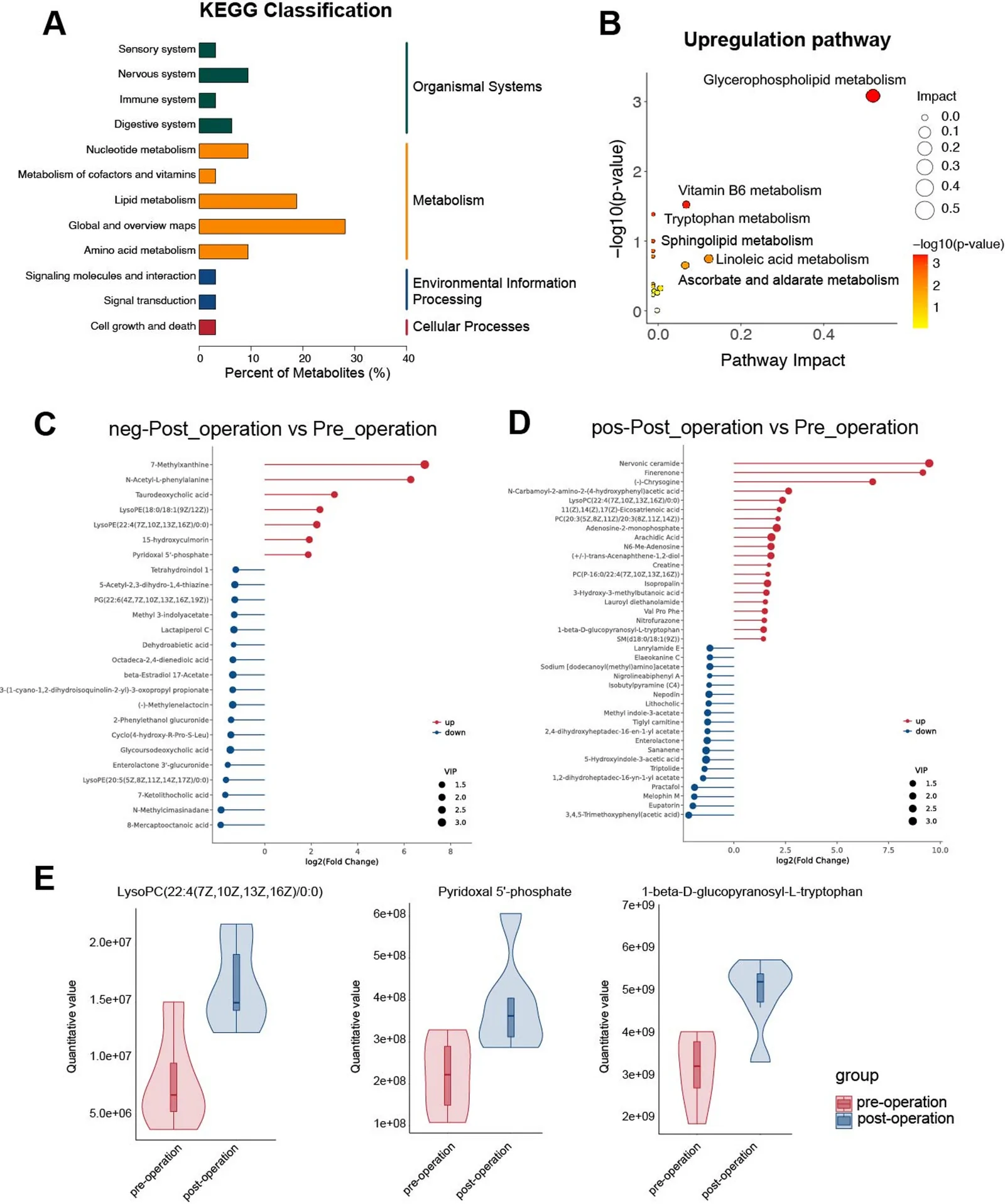
Glycerophospholipid and bile acid metabolism are key pathways altered post-TACE. A KEGG classification of all differentially expressed metabolites, showing major involvement in Lipid metabolism and Amino acid metabolism. B Pathway enrichment analysis of significantly upregulated metabolites. The bubble plot shows the most impacted pathways, with bubble size representing pathway impact and color intensity representing statistical significance (-log10(*p*-value)). Glycerophospholipid metabolism is the most significantly enriched pathway. C, D Lollipop plots highlighting the significant upregulation of LysoPC(22:4) in negative (C) and positive (D) ion modes. E Boxplots showing the significant increase in quantitative values of LysoPC(22:4(7Z,10Z,13Z,16Z)/0:0), Pyridoxal 5'-phosphate and 1-beta-D-glucopyranosyl-L-tryptophan in post-operation samples compared to pre-operation samples.

To elucidate the biological significance of the observed metabolic shifts, we performed pathway analysis on the differentially abundant metabolites. KEGG classification revealed that the altered metabolites were predominantly involved in major metabolic categories, including Lipid metabolism, Amino acid metabolism, and Global and overview maps **(**Fig. 2A**)**. Focusing on the metabolites that were significantly upregulated after TACE, pathway enrichment analysis identified Glycerophospholipid metabolism as the most significantly impacted pathway **(**Fig. 2B**)**. We observed marked increases in specific lysophosphatidylcholines, including LysoPC(22:4) **(**Fig. 2C, 2D**)**, together with changes in bile acid- and tryptophan-related metabolites such as pyridoxal 5'-phosphate and 1-beta-D-glucopyranosyl-L-tryptophan (Fig. 2E). Collectively, these results indicate that the metabolic sequelae of TACE are not confined to the disruption of cell membrane integrity but encompass broader systemic shifts involving bile acid, vitamin, and amino acid metabolism.

## A distinct tumor transcriptomic signature differentiates TACE responders from non-responders

**Fig. 3:**
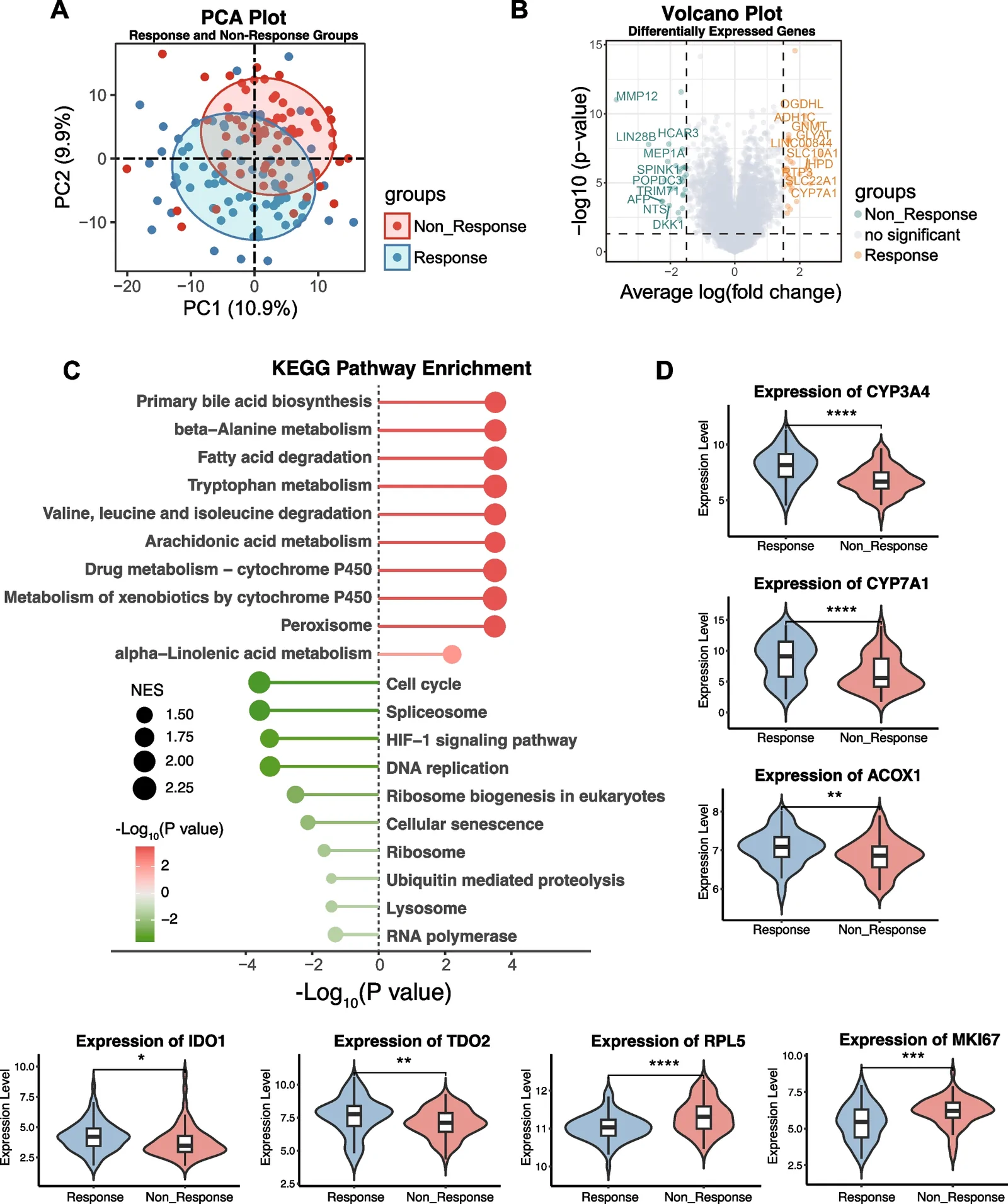
A distinct tumor transcriptomic signature differentiates TACE responders from non-responders. **A** PCA plot of tumor gene expression profiles from the GSE104580 cohort, showing clear separation between TACE responders (blue) and non-responders (red). **B** Volcano plot of differentially expressed genes, with orange and teal dots indicating significantly upregulated genes in responder and non-responder tumors, respectively. **C** KEGG pathway enrichment analysis. Lollipop plot show pathways significantly enriched in responder tumors (top, e.g., Primary bile acid biosynthesis) and non-responder tumors (bottom, e.g., Cell cycle). NES: Normalized Enrichment Score. **D** Boxplots showing the significantly higher expression levels of key metabolic genes (IDO1, TDO2, CYP7A1, CYP3A4, ACOX1) and lower expression of proliferation-related genes (RPL5, MKI67) in responder versus non-responder tumors

To identify the intrinsic tumor characteristics governing treatment response, we analyzed tumor RNA-sequencing data from the GSE104580 cohort. PCA of gene expression profiles revealed a distinct separation between responder and non-responder groups, suggesting a pre-existing transcriptomic program is a key determinant of TACE sensitivity (Fig. 3A). Differential expression analysis highlighted numerous dysregulated genes **(**Fig. 3B**)**. Notably, tumors from responders were characterized by the significant upregulation of genes related to key hepatic metabolic functions, including *CYP7A1*, *ACOX1,* and both *IDO1* and *TDO2* (Fig. 3D). Pathway enrichment analysis demonstrated enrichment of Primary bile acid biosynthesis, Fatty acid degradation, and Tryptophan metabolism, whereas non-responders were enriched for Cell cycle, DNA replication, and Ribosome biogenesis (Fig. 3C). This analysis reveals a fundamental dichotomy in the underlying tumor biology: non-responders are governed by a proliferative program focused on biomass accumulation, while responders prioritize a program of high metabolic activity, characteristic of specialized cellular functions.

## Metabolic signatures are associated with tumor aggressiveness and prognosis in independent HCC Cohorts

**Fig. 4:**
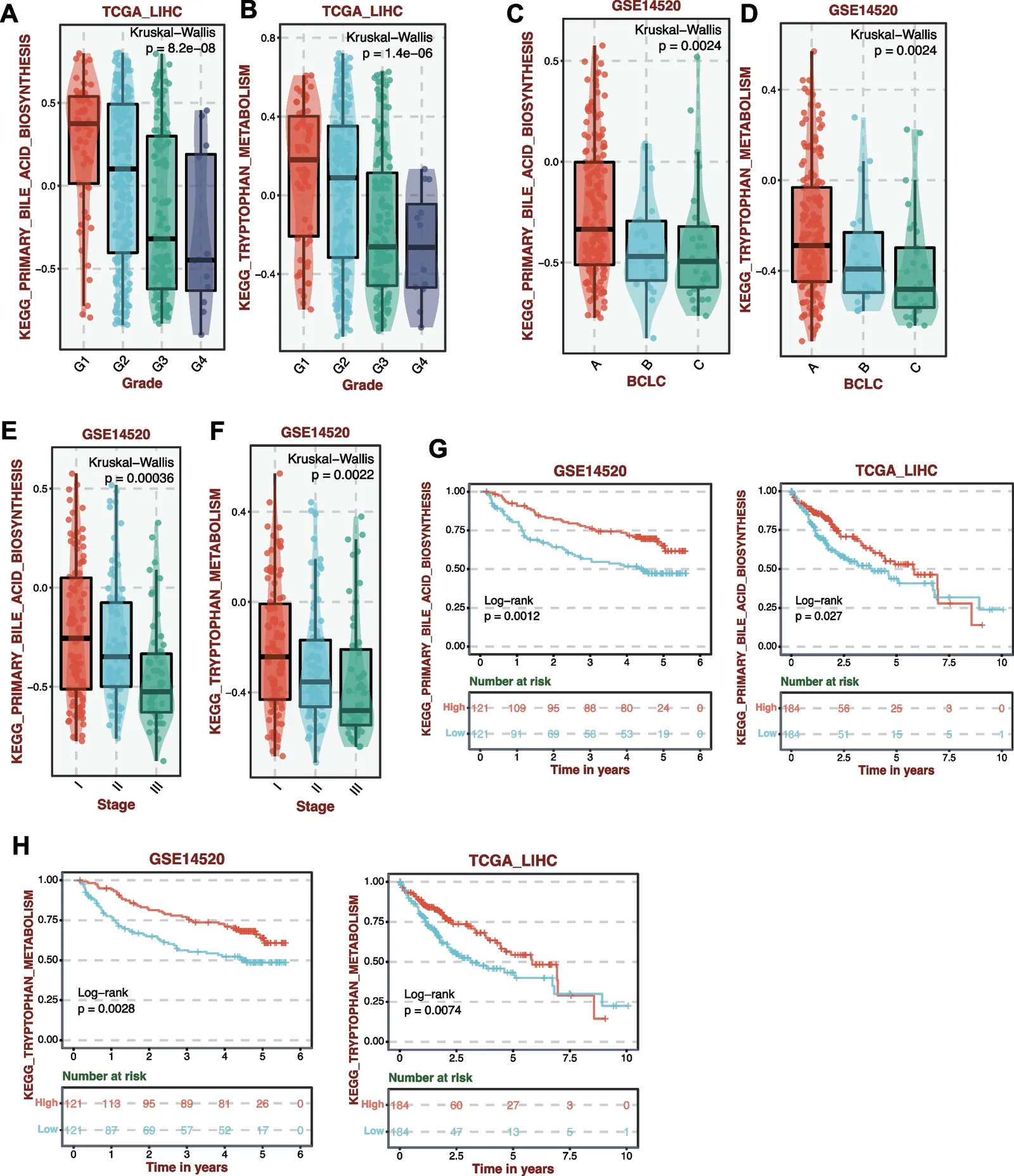
Metabolic signatures are associated with tumor aggressiveness and prognosis in independent HCC cohorts. **A **Primary bile acid biosynthesis pathway enrichment scores across histological grades in TCGA-LIHC. **B** Tryptophan metabolism pathway enrichment scores across histological grades in TCGA-LIHC.** C** Primary bile acid biosynthesis pathway enrichment scores across BCLC stages in GSE14520. **D** Tryptophan metabolism pathway enrichment scores across BCLC stages in GSE14520. **E** Primary bile acid biosynthesis pathway enrichment scores across pathological stages in GSE14520. **F** Tryptophan metabolism pathway enrichment scores across pathological stages in GSE14520. **G** Kaplan-Meier curves for overall survival according to primary bile acid biosynthesis pathway enrichment in GSE14520 and TCGA-LIHC. **H **Kaplan-Meier curves for overall survival according to tryptophan metabolism pathway enrichment in GSE14520 and TCGA-LIHC.

Our validation analysis revealed a significant correlation between the metabolic pathway scores and features of tumor aggressiveness. The enrichment score for both primary bile acid biosynthesis and tryptophan metabolism differed significantly across histological grades in the TCGA-LIHC cohort, with lower scores observed in higher-grade tumors (G3/G4) (Fig. 4A, B). This association with advanced disease was corroborated in the GSE14520 cohort, where both primary bile acid biosynthesis and tryptophan metabolism scores were lower in more advanced BCLC stages (Fig. 4C, D) and pathological stages (Fig. 4E, F). These findings suggest that reduced activity of these metabolic pathways is associated with a more aggressive tumor phenotype. Furthermore, these metabolic signatures demonstrated significant prognostic value. Kaplan–Meier survival analysis showed that high enrichment of the primary bile acid biosynthesis pathway was significantly associated with longer overall survival (OS) in both the GSE14520 and TCGA-LIHC cohorts (Fig. 4G). Similarly, high enrichment of the tryptophan metabolism pathway was also associated with significantly longer OS in both cohorts (Fig. 4H).

## Integrative analysis links tumor metabolism to systemic fecal metabolite changes

The final step of our analysis was a cross-cohort integration of the two omics datasets to construct a cohesive but explicitly hypothesis-generating biological model of TACE response. Because fecal metabolomics and tumor transcriptomics were obtained from separate and unmatched cohorts, the integrative analysis does not establish direct patient-level tumor-fecal crosstalk. Instead, it identifies convergent pathway-level themes across complementary data layers. A notable parallel was observed in bile acid metabolism: responder tumors showed upregulation of the Primary bile acid biosynthesis pathway and the rate-limiting enzyme CYP7A1, while fecal metabolomics revealed increased bile acid-related signals after TACE. Together, these observations support a putative model in which a responder-associated tumor metabolic phenotype is linked to systemic bile acid-related perturbation across the gut-liver axis.

A second parallel was observed in lipid metabolism. Responder tumors exhibited enrichment of fatty acid degradation and peroxisomal programs, highlighted by higher ACOX1 expression, whereas fecal samples showed a pronounced post-TACE increase in LysoPC-related species. Rather than interpreting this pattern as direct proof that fecal LysoPC exclusively reflects tumor necrosis, we now view LysoPC as a composite metabolic signal of acute treatment-induced tissue injury and host–microenvironmental perturbation. Several non-mutually exclusive processes may contribute, including breakdown of membrane phospholipids from dying tumor cells, collateral hepatocyte injury, inflammatory remodeling, intestinal epithelial turnover, and microbial lipid biotransformation. Thus, the cross-cohort integration supports a biologically plausible pathway-level model but remains inferential until validated in matched longitudinal multi-omics cohorts.

## Discussion

Our finding that the Primary bile acid biosynthesis pathway is a hallmark of TACE-responsive tumors is of significant biological and clinical interest. Bile acid synthesis is a highly specialized function of differentiated hepatocytes. Its preservation and upregulation in HCC may indicate a tumor that retains features of a more differentiated, less proliferative phenotype. Such tumors may be inherently more vulnerable to the metabolic and ischemic insults delivered by TACE compared to poorly differentiated, highly proliferative tumors that have shed these specialized hepatic functions in favor of rapid growth [22]. The loss of these functions is often associated with increased chemoresistance in liver cancers [23]. We therefore emphasize tumor metabolic phenotype rather than deterministic causal inference: enrichment of bile acid-related pathways should be interpreted as a responder-associated correlate that may reflect both retained hepatic specialization and potential metabolic vulnerability to embolization-induced stress.

CYP7A1, the rate-limiting enzyme in the classical bile acid synthesis pathway, emerged in our analysis as a candidate tumor-intrinsic correlate of the responder phenotype. Elevated CYP7A1 expression in pre-treatment tumor tissue may therefore mark a biological state associated with greater TACE sensitivity. This observation also provides a plausible link to the gut-liver axis. Tumors with greater bile acid synthetic capacity may contribute more substantially to the enterohepatic bile acid pool, thereby influencing the composition and metabolic activity of the gut microbiota [24]. This raises the possibility that the intrinsic metabolic state of the tumor may shape the gut environment and, in turn, help establish a systemic milieu more permissive to favorable treatment response. An alternative, and not mutually exclusive, explanation is that tumors with high metabolic activity, including active bile acid synthesis, may exhibit a form of metabolic vulnerability that renders them more susceptible to the acute ischemic and metabolic stress imposed by TACE. From this perspective, high CYP7A1 expression may reflect not only a more differentiated hepatic phenotype, but also a metabolically vulnerable state associated with treatment responsiveness. However, the present study was not designed as a formal biomarker-development investigation with independent discovery and validation cohorts. Accordingly, we do not present CYP7A1 as a validated predictive biomarker, but rather as a candidate marker emerging from an exploratory integrative analysis. In addition, because the transcriptomic dataset was independent of the local metabolomics cohort, we were unable to perform joint patient-level modeling, ROC/AUC evaluation, or direct radiologic response analyses integrating CYP7A1 expression with fecal LysoPC levels and imaging outcomes. Further validation in larger, prospectively collected cohorts will be required to determine its predictive and translational value.

A critical and potentially paradoxical finding of our study is the association of higher IDO1 and TDO2 expression with favorable TACE response. In many contexts, particularly immunotherapy, the *IDO1/TDO2* pathway is a well-established mechanism of immune suppression, creating an immunosuppressive tumor microenvironment by depleting tryptophan and producing kynurenine, which inhibits T-cell function [13]. Instead, at least three non-mutually exclusive interpretations may explain the result. First, IDO1/TDO2 upregulation may mark a more differentiated or metabolically specialized tumor state, consistent with the responder-associated increase in CYP7A1, ACOX1, bile acid biosynthesis, and fatty acid degradation pathways. Second, metabolically specialized tumors may be more vulnerable to the acute ischemic, hypoxic, and nutrient-deprived conditions created by embolization. Third, baseline tryptophan catabolism may reflect a context-dependent immune-metabolic ecosystem that interacts with locoregional therapy differently from the highly proliferative biology of non-responders. We therefore frame the IDO1/TDO2 result as correlative and hypothesis-generating.

The increase in fecal LysoPCs following TACE provides a powerful, non-invasive signal of therapeutic effect. We propose that this increase may partly reflect membrane phospholipid breakdown secondary to tissue injury and tumor necrosis, resulting in cellular membrane disruption and the release of phospholipid components. As such, measuring the change in fecal LysoPC levels after an initial TACE cycle could serve as an early pharmacodynamic biomarker of treatment efficacy. This metabolic readout might provide a more direct and rapid assessment of tumor cell kill than conventional imaging modalities like mRECIST, which rely on delayed changes in tumor size or vascular enhancement [24]. However, because TACE may also induce non-malignant hepatocyte injury, inflammatory activation, intestinal epithelial turnover, and microbiota-related lipid remodeling, LysoPC should be interpreted as a composite signal rather than a tumor-necrosis-specific marker. Future studies should correlate LysoPC dynamics with liver injury indices, radiologic response, and microbiome features to clarify its biological source and clinical relevance.

It is crucial to recognize that LysoPCs are not merely inert byproducts of cell death; they are bioactive lipid mediators with complex roles in inflammation, apoptosis, and cell signaling [25]. The specific species we identified as highly upregulated, LysoPC (22:4), contains a polyunsaturated fatty acid (PUFA). Some studies suggest that, in contrast to saturated LysoPCs which are generally pro-inflammatory, polyunsaturated LysoPCs may exert anti-inflammatory or immunomodulatory effects [25, 26]. This raises the intriguing possibility that the type of LysoPC released from dying tumor cells could actively modulate the local and systemic inflammatory response following TACE, potentially influencing subsequent tumor control and immune surveillance.

While this study did not directly measure the composition of the gut microbiota, our metabolomic findings provide compelling indirect evidence of its critical involvement. The significant post-TACE alterations in metabolites known to be heavily modulated by microbial activity-such as bile acids and tryptophan derivatives-strongly imply that TACE induces a state of gut dysbiosis [27]. This is consistent with existing literature, which has shown that TACE can disrupt the gut microbial ecosystem, often leading to a reduction in microbial diversity and a decrease in the abundance of beneficial, SCFA-producing bacteria [27]. Together, these observations support a working model in which tumor-intrinsic metabolic programs, treatment-induced tissue injury, hepatic metabolic remodeling, and gut microbial metabolism may interact across the gut-liver axis. However, our data provide only pathway-level concordance across independent cohorts rather than direct patient-level evidence. The proposed gut-liver axis model should therefore be regarded as hypothesis-generating.

Several limitations should be acknowledged. First, the transcriptomic and metabolomic analyses were performed in two separate, unmatched cohorts, which precluded direct correlation between tumor gene expression and fecal metabolites within the same patient. Thus, the integrative findings represent parallel cross-cohort inference rather than direct evidence of tumor–fecal crosstalk. Second, the role of the gut microbiome remains inferential because no 16S rRNA or metagenomic profiling was performed. Third, metabolomic sampling was limited to a single early post-TACE time point (day 4), which likely reflects an acute pharmacodynamic snapshot rather than a stable long-term metabolic state [13]. Finally, candidate markers such as CYP7A1 and fecal LysoPC were identified in an exploratory framework and should not be considered validated clinical biomarkers. Because the transcriptomic dataset was independent of the local metabolomics cohort, joint patient-level modeling, ROC/AUC analysis, and direct imaging-response correlation could not be performed. Larger prospective matched multi-omics studies and functional experiments will be required to validate the biological and translational significance of these findings.

## Conclusion

In conclusion, this study utilizes an integrative multi-omics approach to provide novel insights into the biological basis of TACE response in HCC. Our data suggest that responder tumors are characterized by a metabolically specialized and less proliferative phenotype, particularly involving bile acid, lipid, and tryptophan-related pathways, while fecal metabolomics captures acute post-TACE tissue perturbations including LysoPC and bile acid-related changes. Because the fecal metabolomics and tumor transcriptomics datasets were derived from separate cohorts, these observations should be interpreted as convergent, pathway-level, hypothesis-generating associations rather than direct evidence of tumor-fecal crosstalk. The study therefore nominates CYP7A1 and fecal LysoPC as candidate biomarkers for future validation, highlights the gut-liver axis as a potentially informative framework for understanding locoregional therapy response, and underscores the need for matched longitudinal multi-omics and mechanistic studies.

## Supplementary Information

Supplementary Material 1.

Supplementary Material 2.

## Data Availability

The data that support the findings of study are available from the corresponding author upon reasonable request.

## Abbreviations

AFP: Alpha-fetoprotein
BCLC: Barcelona Clinic Liver Cancer
FDR: False discovery rate
GEO: Gene Expression Omnibus
GSEA: Gene set enrichment analysis
HCC: Hepatocellular carcinoma
KEGG: Kyoto Encyclopedia of Genes and Genomes
LysoPC: Lysophosphatidylcholine
OS: Overall survival
PCA: Principal component analysis
PLS-DA: Partial least squares discriminant analysis
SCFA: Short-chain fatty acid
TACE: Transarterial chemoembolization
TCGA-LIHC: The Cancer Genome Atlas Liver Hepatocellular Carcinoma
TPM: Transcripts per million
